# Drivers of Cousin Marriage among British Pakistanis

**DOI:** 10.1159/000358011

**Published:** 2014-07-29

**Authors:** Alison Shaw

**Affiliations:** University of Oxford, Oxford, UK

**Keywords:** British Pakistanis, Cousin marriage, Migration

## Abstract

**Background/Aim:**

Why has the apparently high rate of cousin marriage among Bradford Pakistanis been sustained, 50 years since Pakistani migration to Britain began?

**Methods:**

A review of the anthropological literature on Pakistani migration and settlement, British Pakistani marriage patterns and the phenomenon of transnational marriage.

**Results:**

British Pakistanis are diverse in regional origins and social class characteristics, with many Bradford Pakistanis originating from the Mirpur district and northern Punjab. British Pakistani marriages often involve a partner from Pakistan who joins a spouse in the UK. Transnational marriage of first cousins offers relatives in Pakistan opportunities for a ‘better’ life in the West and are important for British Pakistanis for economic, social, cultural and emotional reasons. These processes are also differentially influenced by region of origin and class characteristics in Pakistan as well as by education, employment and locality in Britain. The pattern observed in Bradford may not be applicable nationally.

**Conclusion:**

Further research examining marital decisions over several generations in families differing by social class, region of origin in Pakistan and locality in Britain is necessary to contextualise the findings from Bradford.

## Introduction

Cousin marriage is a complex phenomenon influenced by cultural preferences and by political, socioeconomic and emotional factors, the relative influence of these different factors varying across time and place. Cousins who marry are most often first cousins – the sons and daughters of siblings – but may be other consanguineous kin, that is, related as second cousins or closer, or even people considered to be family but who are not necessarily consanguineous. Usually, parents or other relatives arrange these marriages. Cousin marriage is broadly associated with gender norms that restrict interactions between the sexes and with low levels of female education. To be numerically significant, this marriage pattern also depends on families being large enough for suitable cousin spouses to be available. In this article, cousin marriage refers to first-cousin marriages unless specified, and consanguineous marriage refers to marriages between people related as second cousins or closer.

In Western Europe and North America, cousin marriage is now much less common than it was a hundred and fifty years ago. George Darwin estimated that approximately 4.5% of aristocratic marriages and 3.5% of middle-class marriages were with cousins in mid-19th century England [1]. By the mid-20th century, less than 1% of marriages were with cousins [2]. In North America, the comparatively higher rates of cousin marriage associated with some religious communities and minority populations declined rapidly over the same period of time [3]. In Japan, the rate of consanguineous marriage has declined rapidly in the past 50 years [4]. In the Middle East, there is evidence of a recent decline in consanguineous marriage in countries such as Jordan, Saudi Arabia and Turkey [4]. However, there are reports of no significant change in the Palestinian Territories, and of regional increases in the rate of consanguineous marriage in the current generation in Qatar, the United Arab Emirates and Yemen, despite rapid urbanisation [5, 6, 7, 8]. Nevertheless, the expectation for those countries is that in the next generation consanguineous marriage will decline as a result of the demographic transition to smaller families, which will reduce the number of available consanguineous relatives [4]. In short, consanguineous marriage can be expected to decline as people have fewer children, as households become less directly dependent on land and as women achieve higher levels of education.

Given this expectation, British Pakistani cousin marriage presents a puzzle. Evidence from small-scale studies conducted in the 1980s and the 1990s showed cousin marriage to be more common among young British Pakistani adults than among their parents. A West Yorkshire study found an increase from 33 to 55% in the proportion of first-cousin marriages by comparing the marriages of 100 young mothers in the postnatal wards of two maternity hospitals with their reports of their mothers’ marriages [9]. An Oxford study found an increase from 37 to 59% in first-cousin marriages, by comparing 70 marriages of adult children and grandchildren with data on the marriages of their pioneer-generation parents [10].

These data also suggested an increase over ‘base rates’ for cousin marriage reported from Pakistan. These are difficult to establish because of regional, urban-rural, educational and socio-economic variation. Overall estimates are that cousin marriage occurs at a rate of 38–49% in Pakistan, other marriages occurring with more distantly related or unrelated people [11, 12]. This rate is broadly similar to that reported for the Middle East and there has been no significant change over two decades [11]. A large household and obstetric inpatient survey of seven cities in the Punjab province found that 46.8% of marriages were consanguineous, to relatives such as second cousins or closer [12]. Higher frequencies of consanguineous marriage are associated with rural areas, lower levels of education and younger age at marriage, and lower frequencies are associated with urban areas and education, indicating a class dimension to the practice [11, 13].

Fifty years since Pakistani migration to Britain began, there is no firm evidence of any significant decline in the rate of cousin marriage among the children and grandchildren of pioneer-generation migrants. In fact, recent evidence from Bradford indicates that the rate of cousin marriage has been sustained in the current childbearing generation. Mothers of Pakistani origin accounted for 45% of the 11,396 mothers recruited to the Born in Bradford birth cohort study tracking the health of babies delivered in the Bradford Royal Infirmary between 2007 and 2011. In this study, 37% of the 5,127 babies of Pakistani origin had first-cousin parents, and 59% of these babies had parents who are consanguineous [14]. The consanguinity rate is similar to that reported in Birmingham 20 years ago [15]. It also represents an approximately 10% increase over the reported consanguinity of the babies’ maternal and paternal grandparents [16]. By contrast, a recent study from Norway demonstrates that, after an initial slight increase, the rate of first-cousin marriage among Pakistanis declined dramatically from 48.3 to 18.8% over a decade, and there was a 25% increase in marriage to unrelated partners [17]. Why, then, should the practice of marrying relatives be as common, in Bradford at least, as it was a generation ago?

## Pakistani Migration to Britain

Pakistanis now comprise over 2% of the UK's 63.7 million people [18]. They are residentially concentrated in the conurbations of London, the Midlands, the North and the North West. Pakistanis in Bradford account for 20.4% of the total Bradford District population of 522,452 and 9.6% of England's total Pakistani population [19]. They have diverse regional, socio-economic, educational and urban-rural backgrounds in Pakistan, distinctions that are difficult to specify because of rapid urbanisation in Pakistan and because most Pakistani *birādarīs* (extended kinship networks) span villages, cities and national borders. Overall, however, approximately two thirds of British Pakistanis are of rural peasant-farming backgrounds in the *bārān*ī (rainfall-dependent) districts of Mirpur in Pakistan-held Kashmir and Jhelum, Attock, Rawalpindi and Gujrat in northern Punjab [20]. Other British Pakistanis originate from the irrigated and agriculturally more prosperous districts of central Punjab such as Faisalabad, Sahiwal and Sargodha, from Khyber Pukhtunkhua, and from the long-standing city-based educated and professional middle class [21].

Some Mirpuri ex-seamen had settled in Britain in the 1930s, and worked in British factories and foundries during the Second World War, but substantial Pakistani immigration to Britain dates from the 1950s as a direct response to post-War labour shortages in the industrial areas of the Midlands and London, and in the former textile towns of Yorkshire and Lancashire [20]. Migrants were men primarily from areas of Mirpur and northern and central Punjab which have a history of migration and strong historical links with Britain that date from the colonial period. The Punjab of pre-Partition India was a province of British India since 1898, and the British had favoured men from landowning Punjabi castes as recruits for the Indian army, the Royal Indian navy, and as labourers in East Africa [20]. The British also allocated plots of land to peasant farmers from densely populated areas of eastern Punjab, in what is now Indian Punjab, under a scheme to settle the formerly barren ‘canal colony’ districts now central to Pakistan's Punjab [21]. Further internal migration occurred in the exchange of refugee populations at Partition in 1947, as Muslims moved west, to Pakistan, and Sikhs and Hindus moved east, to India.

The first Pakistani labour migrants from Mirpur and Punjab were those who had some prior connections with Britain. As British subjects under the 1948 British Nationality Act, these men had unrestricted right of entry, return and re-entry [20]. They took up work in the factories and mills of the Midlands and the North, returning to Pakistan after long intervals, sometimes risking their jobs in Britain in the process [20]. Their intention was not to build new lives in Britain but to earn money to improve the socio-economic position of families ‘back home’ and eventually to return permanently. The attraction of working in Britain was economic: a labourer's wage was then over 30 times higher than the wage for similar work in Pakistan [22]. Men lived frugally in Britain to maximise the money they could remit [21].

In Pakistan, in the absence of state welfare it falls to the family to provide for all those within it, especially the elderly, those who are ill, children and women. ‘The family’ in a household context is an interdependent and multigenerational unit, comprising parents, sons and their wives, unmarried children and grandchildren. Conventionally marriage is patrilocal: a woman moves to the household of her husband's parents after marriage where her husband and his kin provide for her. Unmarried women are financially dependent on natal kin, particularly fathers and brothers, who are responsible for ensuring she is married and provided for, and a woman can expect support from her father, brothers or sons in the event of marital difficulties, divorce or widowhood. Within the household, obligations to provide support and care are distributed according to gender and hierarchies of age and generation. Men are financial providers and women are responsible for day-to-day care; senior men make major financial decisions and senior women have authority over daughters and daughters-in-law in domestic matters. Expectations of support governed by principles of gender, age and generation also extend to the wider kinship network (*birādarī*) [21, 23].

In migrants’ villages of origin, it was soon apparent that households with wage earners abroad were becoming wealthier than those without, and this fuelled the desire to migrate. Remittances enabled *ghar vāle* (‘people of the home’) in Pakistan to build better houses, buy imported electrical goods, extend landholdings, to buy better agricultural equipment for working the family land and to pay for medical expenses. Remittances were also used to provide good dowries (*jahez*). Dowry accompanies a bride when she moves to her husband's household and typically includes household goods, clothing, jewellery and cash; it also signals the status of the bride and her family, constituting a form of guarantee that a daughter will be properly cared for in her in-laws’ household. Migrants’ relatives also used remittances to sponsor the migration of other kinsmen. Consequently, a pattern of chain migration developed, in which men joined kinsmen in Britain and sponsored the migration of other relatives, underpinning the regional and often village-specific characteristics of many Pakistani settlements in Britain today [20, 21].

### Family Reunification

The pattern of labour migration changed against a backdrop of increasingly restrictive immigration controls, rising unemployment and racial tension. The 1962 Commonwealth Immigrants Act curtailed the entry rights of British subjects from Pakistan. In the 18 months before it was passed, the influx of immigrants seeking to ‘beat the ban’ included men from a wider range of backgrounds than the original labour migrants. After 1962, primary male immigration was restricted to migrants with vouchers, but wives of men already in Britain were permitted to join their husbands and to bring any dependent children, provided these children were under 18 years and accompanied by their mothers. This was also a means of further restricting male labour migration, since some families had been sending boys to join fathers in Britain, to start work as soon as they had left school [20].

Families in Pakistan who had not intended that wives and children would migrate now opted for ‘family reunification’ under immigration regulations that classified men as ‘workers’ and women as ‘dependents’. The regulations for family reunification were changed five times between 1968 and 1983 in order to restrict the immigration of male spouses of Black and South Asian British citizens [24]. Often there were long delays in the process. Families had to decide which children would emigrate and which would remain in the care of relatives. The absence of formal birth records in parts of rural Mirpur and northern Punjab caused complications when family reunification applications were – and continue to be – carefully scrutinised for documentary proof of relationships to applicants before entry visas were issued. Unsuccessful applications sometimes resulted in years of delay while applicants appealed against immigration decisions.

Nonetheless, the number of Pakistani women and children increased significantly from this point onwards, and members of the ‘reunited’ family – according to the British state's definition of it as comprising a man, his wife and their dependent children – were then entitled to apply for British citizenship. The process of family reunification continued through the 1970s and 1980s, with the overall effect of transforming the earlier transitory allmale settlements. With the arrival of women and children from Pakistan, combined with the births of children in Britain, the British Pakistani population increased from 25,000 in 1961 to 119,000 in 1971, more than doubling again in the following decade [25].

In Britain, Pakistani wives who had previously been living in extended households now had opportunities to establish their own homes and this helped re-establish significant features of Pakistani family life in the new environment. Conforming to the requirements of purdah, wives generally did not seek paid work outside the home but established social networks with other Pakistani women, drawing on ties of kin and village in Pakistan created by chain migration. Women's networks provided opportunities for shared religious activities and celebrations of Islamic festivals, also sharpening migrants’ concerns to protect their children from the potentially corrupting influences of Western society. Qur'an classes were established for children at local mosques, which by the 1980s had been established in most towns where Pakistanis settled [21, 26].

These families were not in every case ‘reunited’ even according to the British state's narrow definition of family. From the outset, migration to Britain separated men from their parents, wives and children. Family reunification’ brought wives and children to join men in Britain, but simultaneously separated other closely related people. Older children who were not entitled to migrate were separated from their mothers and siblings and left in the care of relatives, while wives who migrated were separated from their parents and siblings, as well as from their inlaws and older children who remained in Pakistan. It is thus difficult, if not impossible, to separate the spheres of family in Britain and family in Pakistan, and more appropriate to speak of the British Pakistani transnational family. Family reunification also had the effect of increasing the expectations of kin who had remained in Pakistan that they too would continue to benefit from having an outpost of the *birādar*ī in Britain, and from having supported the migration. In some households, those who had remained working the family land or struggling to make a living were sometimes bitter that their children would lack the opportunities now available to nephews and nieces in Britain. Family reunion thus increased inter-family tensions arising from those perceived to have directly benefitted from migration and those who had not [21].

It also did not remove pressures on migrants in Britain to remit money to support relatives in Pakistan. In some cases, it increased these pressures because migrants now had less money available to remit and more expenses in Britain. Migrants continued, to varying extents, to provide for relatives in Pakistan and to help ensure daughters, sisters and nieces were respectably married. Migrants have remained in regular contact with relatives in Pakistan, using the now numerous cheap air routes between Britain and Pakistan to visit kin and attend weddings and funerals. In these on-going contacts, migrants are not merely visitors but are also, to varying extents, active participants, most directly in the matter of arranging marriages and sponsoring the immigration of spouses from Pakistan.

### Spousal Immigration

Since the 1980s, what is in effect, a new phrase of immigration began and has continued to the present day, involving the immigration of spouses from Pakistan who have married British citizens. In the 1980s and early 1990s, when the older Pakistan-born children of pioneergeneration migrants reached marriageable age, their parents usually arranged their marriages to Pakistan-born spouses, who then joined their British-born partners. Analysis of statistics from Bradford estimated that 57.6% of Pakistani marriages during the period 1992–1994 were to spouses from Pakistan [27]. In Oxford in the late 1990s, 50 (71%) of 70 marriages of ‘second generation’ – but not all British born – adult children of 24 pioneer-generation couples were to spouses from Pakistan [10]. From the 1990s onwards, British citizens marrying from Pakistan in this form of family reunion have increasingly been the British-born children or grandchildren of pioneer-generation migrants. Immigration statistics indicate that approximately half of British-born Pakistanis aged 19–50 years has a spouse who has migrated to Britain; according to the 2001 Census, a majority of Britain's Pakistani population (487,000 of 787,000) is Pakistan-born [28].

In Western Europe, spousal immigration is a common consequence of transnational marriage within minority groups originating outside Western Europe [29]. Typically, a European citizen of minority background marries in their country of origin and their spouse joins them as soon as entry clearance from immigration authorities is obtained [30]. These marriages have diverse forms and functions [29, 31]. Some transnational marriages are individually contracted between citizens of different countries who meet while one or both partners are studying or working abroad. In Europe, the largest proportion is marriages ‘arranged’ by families or brokers, and occurs when people who obtain citizenship abroad return to their countries of origin for the specific purpose of marriage. The literature documents the role of arranged transnational marriage in the creation and reproduction of internationally connected South Asian networks: among, for example, British Gujaratis, the Sindhi diaspora and other Indian trading communities, including the wealthy and educated transnational techno-capitalist class of Indian entrepreneurs in Silicon valley [32, 33, 34, 35]. This form of transnational marriage must be understood in the context of global inequalities enabling, among their other motivations, citizenship in the West for the incoming spouse. This can give families based in Europe considerable status and bargaining power in the marriage markets of their countries of origin.

In Britain, spousal immigration is currently the largest single category of immigration for settlement. It is also characteristically South Asian: India, Pakistan and Bangladesh provide one third of all spouses granted settlement, and Indian and Pakistani spouses constitute the two largest groups by country of origin [29]. This pattern, a consequence of British post-colonial labour migration and family reunification, represents family-based strategies for maintaining connections with the subcontinent and expanding the migrant network abroad. However, there is increasing diversity in types of marriage migrants, for example, ‘highly skilled science-oriented migrants’ from India are granted spousal settlement [29].

Analysis of spousal settlement data from the period 1993–2008 shows that wives comprise the majority (60%) of spousal migrants to Britain overall, but there is considerable variation in gender ratios by country of origin: 93% of Thai and 84% of Chinese spouses are wives [29]. The gender ratios for South Asian spouses are more balanced: in 2008, wives comprised 54% of Indian, 56% of Pakistani and 54% of Bangladeshi spouses [29]. This gender balance represents a striking inversion of the South Asian pattern of patrilocal marriage, whereby a woman traditionally joins her husband's household. In the ‘overall upward trend in grants of settlement to spouses’, immigration regulations have been particularly aimed at South Asians, with the aim of preventing forced marriages and marriages for immigration purposes. The Primary Purpose rule, in force since the early 1980s, required foreign nationals married to British citizens to prove the marriage was not primarily for the purpose of obtaining British residency. The applicants had also to prove they could support their spouse ‘without resort to public funds’. These requirements made it particularly difficult for South Asian women to bring husbands to Britain; after the Primary Purpose rule was abolished in 1997, the number of spousal entry clearance applications for men increased and the proportions of incoming husbands and wives are now about equal [29].

Transnational South Asian marriage practices have also resulted in both continuity and transformation of the traditional power and gender dynamics of South Asian marriages. South Asian men have expressed a preference for brides from the subcontinent, thinking they will make more subservient, less assertive wives, and be more effective than UK-raised women in transmitting cultural and religious values to their children [32, 36]. At the same time, incoming wives stand to gain considerable status and bargaining power within the *birādar*ī ‘back home’ in the matter of arranging transnational marriages for their children and potentially facilitating the migration of relatives from South Asia. A ‘feminisation of migration’ has been noted within other diasporic South Asian groups, such as middle class Sikh Jats in Canada, whose marriage strategies construct women as ‘agents of marital citizenship’, with transformative effect on practices and norms concerning gender and status [37].

British Pakistani women have expressed a preference for husbands from Pakistan, considering such men are likely to have a better knowledge of Islam and Pakistani cultural norms than British-raised Pakistani men. However, such marriages also offer opportunities for shifting power relations in marriage in favour of women, undermining a man's ability to dominate his wife [30]. An ‘imported’ son-in-law is traditionally in a weak position in the household of his wife's parents, representing a mirror image of the traditional position of a daughter in her husband's household (the term *ghar damad,* literally ‘son-in-law of the home’, is derogatory, as it implies the man cannot support his wife) [38]. In Britain, an immigrant husband is directly dependent on his wife and her household until residency is granted. This shift in gendered power relations may be a legacy of the greater autonomy exercised by pioneer-generation wives in their households in Britain, free of the authority of their mothers-in-law [21].

British South Asian marriages are usually ‘arranged’ or ‘semi-arranged’ [29]. British Indian Sikh and Hindu marriages tend to be arranged within the caste and may include sibling exchange marriages (whereby two brothers marry two sisters, or a brother and sister marry another sibling pair), but marriages between people with whom a kinship link can be established according to customary rules of exogamy are prohibited [39]. By contrast, although some British Pakistani transnational marriages are to unrelated spouses and some cousin marriages occur between UK-raised spouses, the available published data indicate that most British Pakistani transnational Pakistani marriages involve consanguineous kin. In Oxford in the late 1990s, 46 (92%) of 50 transnational marriages were with kin, 37 of them with first cousins and 9 of them with other relatives [10]. In a recent study of 51 Mirpuri and Punjabi couples aged 20–43 years referred between the period 2000–2004 to a genetics clinic in High Wycombe, 47 (92%) of the couples were consanguineous, 36 related as first cousins, and in all but one of the couples one partner was a marriage migrant [40]. What, then, is the significance of cousin marriage for transnational Pakistani families?

## Pakistani Marriage Choices: Strategy and Emotion

As in other parts of South Asia, the Middle East, and North Africa, marriage with a relative such as a first cousin is preferred [3, 41, 42]. A common justification for this is that you know your relatives, so a daughter's transition into her husband's household will be easier if she is married into a family of known, trusted kin. In this respect, a first-cousin marriage is often considered an ideal match – as a match between the closest relatives beyond the circle of kin is prohibited by incest rules. This customary preference is supported by Islamic marriage rules: Islam, like Judaism and Protestant Christianity, excludes first cousins from the list of relatives forbidden as spouses [4, 43].

A consequence of preferential close-kin marriage is that Pakistani marriages tend to be endogamous to the *birādarī.* In Pakistan, *birādarīs* have names that derive from traditional occupational status groups or castes *(zāt)*. The numerous *birādarīs* and their subgroups fall broadly into three hierarchically ranked categories: *ashraf* (noble, respectable), *zamīndār* (landowning) and *kammī* (artisan). Families vary in the importance they attach to *birādarī* endogamy in actual marriage choices. While high-ranking families may be especially concerned to maintain caste identity, landowning families may be more concerned about the relative status of different *birādarīs* within the overall caste *(zāt)* hierarchy. For some families, marriage across *birādarī* boundaries offers a form of social mobility, for example, where this is connected with cementing a connection between business partners [21, 44].

Besides caste identity, many other interests are at stake in actual marriage choices. Marriage choices in practice are not just a matter of following a cultural preference but involve an assessment of many different factors as parents seek an appropriate and desirable match for their son or daughter. Potential spouses outside as well as within the *birīdarī* may be considered. Parents usually assess the reputation, economic standing and personalities of the potential in-laws and the educational level and occupation of the potential groom or bride. Strategic marriage choices enable social mobility even within the extended kinship network. *Birādarīs* themselves may sub-divide along socio-economic and class lines when intermarriage occurs only between families of similar income, educational and professional status, and proposals (usually initiated by the boy's family) from families considered inferior in these respects are turned down [21, 45].

The socio-economic and emotional interests of a range of actors – parents, extended family and the young people themselves – may all become relevant, to varying extents, when *rishte* (connections through marriage) are considered. Particular choices are often the outcome of negotiations between parents and other members of the extended family. If a father desires a FBD (father's brother's daughter) match for his first son, the mother may wish a MBD (mother's brother's daughter) match for the second son. Sometimes, too, marriages that have proved successful may be followed by other marriages linking the same two households. To understand a particular choice it is necessary to understand the socio-economic circumstances and all the relationships – historical and current – between those involved [41, 45].

Especially for transnational families, marriage choices are also strongly underpinned by emotional connections between kin separated by migration. Transnational arranged marriages within the *birādarī* have enabled some older children in Pakistan to be reunited with natal kin in Britain, while first-cousin marriages enable siblings separated by migration to reconnect through the marriages of their children. Moreover, these marriages are nowadays rarely a matter of parents in Britain insisting on an appropriate match for their child on the basis of their own desires for alliances with particular siblings or to protect property or land in Pakistan. In choosing among nieces or nephews of marriageable age, parents also take account of the compatibility of potential spouses and the desires of young people themselves. There is awareness across the generations of the distinction between forced and arranged marriages, with young people playing a larger role in the process of spouse selection. As a result, particular engagements may have to be broken off and reconsidered, risking rifts in relationships between transnationally divided kin [43].

Cousin marriage has been analyzed as a particularly effective means of ensuring ‘cultural continuity’ through its mechanisms of intergenerational transmission of practices and values [3]. Young British Pakistanis in Bristol and Oxford have spoken of the advantages of marrying someone who is known to their family, and who understands the social norms, language and religion of Pakistan. These cultural considerations could extend to the choice of unrelated transnational spouses, but the concept of a transnational ‘cousin’ carries connotations of both closeness, as someone ‘from the family’ and distance, which makes them more acceptable to British Pakistanis than local cousins, who are seen as ‘too close’ to be considered as spouses [46]. Young British Pakistani adults have spoken of their initial meetings with and subsequent engagements to their Pakistani spouses in both modern and distinctly South Asian romantic imagery, describing how they negotiate these relationships through international phone-calls or chaperoned outings during visits to Pakistan [46].

These views suggest strongly that Pakistani arranged transnational cousin marriages do not conform to a purely socio-economic strategy, as a straightforward continuation of labour migration or a route to residency and citizenship in Britain, but indicate intergenerational changes in the motivations for these marriages. Within the British Pakistani population, there is also awareness that even a first-cousin marriage, which is traditionally considered the ‘least risky’ especially for a daughter, carries new risks when the marriage is transnational [47]. There is the possibility that a spousal entry visa will be rejected and a married daughter may in effect become a single parent; another risk is that the incoming spouse (and their family in Pakistan) is using the British spouse as a ‘passport’ to the West without any commitment to the marriage [10, 47]. Transnational marriages of people raised continents apart, even when they are first cousins, can also fail for reasons that include spousal incompatibility and conflicting expectations of the marriage [10, 48]. Conflict over the use of an incoming husband's wage where husbands expect to remit money to Pakistan may also contribute to marital instability [43, 48]. Changing expectations of marital intimacy, in its modern sense of personal fulfilment and companionate marriage, are also an increasingly important factor in destabilising marriage. From this perspective, some young people and their parents are viewing arranged marriages as ‘riskier’ than love marriages. Islam is emerging as a significant resource for some young women in asserting their rights, including rights to divorce [48].

## Regional and Social Class Effects

Differences exist within the British Pakistani population in the relative weight of these motivations for cousin marriages. These differences are linked to social class and aspects of regional origin in Pakistan, combined with regional and locality-specific characteristics of Pakistani settlements across the UK. Large-scale survey data show the British Pakistani population overall to be almost the poorest and most socially marginalized of Britain's South Asian minorities – the most economically marginal group being Bangladeshis mainly from the Sylhet region of northeast Bangladesh [49]. Pakistanis and Bangladeshis have the lowest percentages of people in higher managerial professions, the highest percentages of people who have ‘never worked or [are] long-term unemployed’ (16.2% of Pakistanis compared with 2.7% for the whole population) and the highest percentages of people with no educational qualifications [49]. They also have the lowest rates of female participation in the labour force: only 22% of Bangladeshi women and 29% of Pakistani women aged over 16 were economically active, compared with 55% of Indian (Hindu and Sikh) women. Pakistani and Bangladeshi women have also on average more children than women from other ethnic groups [49]. Many British Pakistani households are large but have just one wage earner, or have no wage earner and are dependent on state benefits.

National data on region of origin have not been collected, but local and anthropological studies indicate that a majority of British Pakistanis are from the remote Mirpur district in Pakistan-administered Kashmir and parts of adjacent Jhelum [20]. They are residentially concentrated in some of the poorest inner city areas of Birmingham in the Midlands and Bradford and Leeds in Yorkshire. In the Born in Bradford study, two thirds of the study participants were from the most deprived households in the UK [14]. The proportion of Mirpuris is sometimes overestimated: one report states that in cities such as Bradford and Birmingham ‘up to 90% of the Pakistanis have their roots in Mirpur’ [50]. By contrast, a report on the Born in Bradford study suggests a lower proportion, stating that ‘almost half of the South Asian population of Bradford is of Mirpuri origin’ [51]. Different definitions of ‘roots’ and ‘origins’ and of Mirpuri, Kashmiri and Punjabi identities are likely to account for some of this discrepancy. There are also smaller Mirpuri/Kashmiri settlements closer to London, in Luton, Slough, Maidenhead and High Wycombe, where local estimates suggest that approximately half of the Pakistani population is of Mirpuri origin [43]. Emigration from the Mirpur district accelerated in the 1960s when over 280 villages and the old towns of Mirpur and Dadial were submerged for the construction of the Mangla Dam. More than 110,000 people were displaced, some moving first to the adjoining Jhelum district. The Pakistan government promoted the migration of 5,000 of those who had lost their land [52]. Many of those displaced joined relatives in Britain, and some received work permits for the UK

Processes of family reunification were particularly slow among British Mirpuris, many of whom were unaccustomed to the bureaucracy of the immigration process. Mirpuri family reunification also coincided with a contracting economy in Britain [20, 39]. In Bradford and Birmingham, Mirpuris were among those hardest hit by the recession of the 1980s and 1990s. Detailed research on the socio-economic trajectories and current remittance patterns of Pakistani families of different social classes and regional backgrounds in the UK is lacking, but it is likely that the disposable income available to households in Britain continues to be depleted by the need to remit at least some money to kin in Pakistan.

Routes for social advancement in Mirpur itself have been restricted to the construction and service industries meeting the needs of visiting migrants [39]. British Mirpuris have spent money on items of conspicuous consumption: the palatial *kotīs* (bungalows) of families in Britain are striking landmarks in the rural areas, signalling the status of families abroad, but they often stand empty for long periods, with poorer relatives as caretakers [50]. Some pioneer-generation men have retired to these houses, living on their pensions from England, but their wives and adult sons have remained living in Britain, returning only for weddings, holidays and to see relatives. In this region, the lack of alternative strategies for social mobility means that marriage to relatives in Britain continues to be viewed as the main route to a better life [39].

In High Wycombe, where I conducted fieldwork during the period 2000–2004 [43], I observed that Mirpuriextended families have sometimes settled in many houses on the same streets of run-down estates, in effect creating working class ‘ethnic enclaves’ characterized by dense networks of kin who have relocated in Britain. In this context, parents arranging a daughter's marriage to a cousin from Pakistan may view the new son-in-law as someone who will help boost the income of the UK-based family, even though the husband may expect or feel under pressure to remit money to Pakistan. Spousal immigration for working class families in deprived parts of Britain may thus entail a struggle for short-term control of wages rather than a long-term strategy of transnational citizenship.

I have described a ‘working-class Mirpuri’ pattern for heuristic purposes, not because all Mirpuri families follow this route. This pattern can be contrasted with the socio-economic strategies of families from central and other parts of Punjab, and those of longer-standing ‘middle-class’ origins, where there is more emphasis on transnational business or education. A central Punjabi pattern seems to characterize British Pakistanis of middle-ranking landowning families from central Punjab districts, such as Faisalabad, who have established a niche for themselves in family-run wholesale and retail enterprises in, for example, Manchester, Rochdale, Oxford and Glasgow. In Manchester, an urban educated Pakistani middle class that includes representatives of Pakistani banks and other commercial interests has influenced social dynamics in the city [44]. Faisalabadi families in Oxford have generally drawn on the labour of the extended family in Britain to expand their businesses and remit money to Pakistan where they have invested in projects, such as chicken farming, buffalo milk businesses, ice factories and construction, in their villages or origin and in Faisalabad city. Transnational marriages have been integral to many of these networks, and are usually between cousins but sometimes link unrelated kin. In general, these families have a more outward looking and opportunistic approach to marriage, mediated by *zat* status [21].

An emphasis on business and education can offer opportunities for returning to Pakistan that are unavailable to working-class families, as in the following examples, updated here from my earlier fieldwork in Oxford [21]. Mujeeb is from a middle-ranking landowning rural family from the Attock district who became a successful retailer and arranged the marriage of his university-educated daughter to the son of his business partner in Pakistan. His daughter has lived in Rawalpindi since her marriage, returning to the UK for the births of her children, who all have British citizenship. Munir is from an *ashraf* family in the same village. His older son followed his father as a blue-collar worker, but the younger son obtained a university degree and then established a property rental business. Both sons married first cousins who came to the UK from Pakistan. While the elder son's children are now also married to cousins, one daughter did not apply for a spousal visa for her army-officer husband, but instead moved to Pakistan after her son was born. Munir's second son has also moved with his wife and children to Pakistan, leaving the day-to-day aspects of the business in the hands of his UK business partner. He has established a business in Pakistan close to his in-laws’ home, and his children attend English-medium schools in Pakistan; the eldest child has recently returned to Britain in order to study law at university, so he will have transnational options for a professional career.

These strategies of education and business among originally village-based families share characteristics with the long-standing internationally connected expatriate Pakistani urban middle class. This class has less need to remit money to Pakistan, and is better placed to invest in property, business and education. In such families, lives are transnational in that they are lived part of the time in Pakistan and part of the time abroad, according to need and opportunity. For example, to take another case from my recent fieldwork, the British-born, university-educated daughter of a Karachi-based Punjabi family that is also settled in High Wycombe has recently moved with her school-age children to Karachi, where her children will be educated in English-medium schools, in order that her husband (her father's brother's son) can join his brother in business there. This woman's brother, by contrast, is currently establishing a professional career in the city of London, commuting daily from High Wycombe where he lives with his wife (his father's sister's daughter), who is a graduate from Pakistan.

## Discussion

Class and regional effects may also play a part in explaining why, in contrast to Bradford, Pakistani consanguineous marriage in Norway appears to have decreased from 48.3 to 18.8% over 10 years. In a sample of 2,000 Norwegian Pakistani women, cousin marriage decreased most significantly in marriages of Norwegian-born Pakistani women, and was more likely to persist over time where the woman's parents were also consanguineous. The authors attribute this to the continuity of ‘family tradition’ in those households where parents make the main marital decisions, but they do not report in any detail on the social class or regional characteristics of the population, or the proportion of transnational marriages in the sample [17].

Norway's Pakistani population of 30,000, less than half the size of Bradford's Pakistani population, originates mainly from Kharian in the Gujrat district of Punjab. This area is sometimes called ‘Little Scandinavia’ because so many families in this area have relatives in Norway and Denmark, as a consequence of male guest working in the 1960s (after labour migration to Britain was curtailed) and then family reunification after labour-related immigration was curtailed [53]. An anthropological study of Danish Pakistanis indicates some important differences between the UK and Denmark in the characteristics of their respective Pakistani populations. Danish Pakistanis number about 25,000 (not much more than one third of Bradford's Pakistani population) and have followed strategies of competitive moneymaking and investing in business and education [53].

Among Danish-born Pakistanis, connections with non-kin based on educational or religious affiliation are now often extending into choice of marriage partner, with marriages increasingly being contracted within Denmark [53]. This has coincided with a strict family reunification policy, in force since 2002, in which ‘consanguineous marriage’ was defined as forced on the ground that marrying within the family must entail a degree of coercion. One unexpected consequence of this policy is that hundreds of Danish Pakistanis move to Sweden to obtain family reunification with a Pakistani spouse [53]. The proportion of transnational marriages among young Danish Pakistani adults (reported in 1989 as over 80%) has dropped to 59% by 2004; this figure suggests that transnational marriages continue to be important among middle-class Danish Pakistanis even if they are not necessarily with cousins [53].

Besides the regional and class effects, generational differences also influence motivations for consanguineous marriage and, potentially, consanguinity rates. These in turn are not straightforward but mediated by migration history and aspects of immigration policy. The so-called ‘second generation’ descendants of pioneer migrants in Europe are not all European-born but include Pakistan-born adults who migrated as children and subsequently married partners from Pakistan. In a sizeable proportion of Bradford Pakistani couples of reproductive age, both partners are Pakistan-born, as a result of delayed family reunification among Mirpuris and delayed spousal immigration. In other words, the Bradford pattern may be a particular consequence of the migration history, combined with local and regional socio-economic effects, rather than an indication of more deeply entrenched ‘tradition’. The Born in Bradford birth cohort study collected data on birthplace and age of entry to the UK of mothers and fathers of Pakistani babies; it would be instructive to examine consanguinity of couples according to these factors [51].

## Conclusion

Marrying ‘in the family’ is traditionally preferred in Pakistan. Within transnational British Pakistani families, marriage plans for children have become inextricably entwined with possibilities for emigration, not just as a strategy to continue labour migration despite increasingly restrictive immigration controls, but as a route to sharing with other members of the family the benefits and opportunities of life in the West, such as earning potential, education and citizenship, and expanding international opportunities and networks. For British Pakistanis across the generations, marriage to a spouse from Pakistan is also a practical, symbolic and emotional link to the culture and religion of the homeland. Migrants who arrange at least some marriages of their sons and daughters to cousins in Pakistan are continuing to fulfil their commitments to relatives in Pakistan, and gain status and bargaining power within the transnational *birādarī.* Transnational marriage is a form of social mobility for cousins chosen as spouses, marking out their households in Pakistan from those with no such overseas connection.

I have also highlighted social class, region of origin, local and generational effects on the functions and meanings of cousin marriage across this transnational arena. We do not know if the recent findings from Bradford are specific to Bradford, or apply more generally to working-class families of Mirpuri origin in other areas of deprivation and high unemployment in Britain. In cities such as Manchester and Oxford, with significant proportions of Pakistanis from other parts of north, central, rural and urban Punjab, the current prevalence of cousin marriage may be lower, even though transnational marriage may remain important. A comparative longitudinal study examining marital decisions and processes of family reunion over several generations in families differing by social class, regional origin and locality in Britain would be necessary to answer this question. Historical data suggest that maintaining close family networks through close-kin and first-cousin marriage is important under conditions of socio-economic insecurity, for example, in the early 19th century in Europe, where families faced high risks during the upheavals of early industrialisation [43]. In some areas at this time there were local increases in several types of close-kin marriage, including marriages of first cousins, even though by the end of the 19th century the rate of cousin marriage had declined to its present low levels [54, 55, 56]. A parallel argument could be made for consanguineous marriage within minority Muslim communities in the contemporary world.
